# Cystic fibrosis – a multiorgan protein misfolding disease

**DOI:** 10.4155/fso.15.57

**Published:** 2015-09-01

**Authors:** Douglas Fraser-Pitt, Deborah O’Neil

**Affiliations:** 1 NovaBiotics Ltd, Cuickshank Building, Craibstone, Aberdeen, AB21 9TR UK

**Keywords:** bacterial biofilms, CFTR, cystic fibrosis, lung infection, *Pseudomonas aeruginosa*, respiratory disease

## Abstract

Cystic fibrosis (CF) is a heterogeneous multiorgan disease caused by mutations in the *CFTR* gene leading to misfolding (and other defects) and consequent dysfunction of CFTR protein. The majority of mutations cause a severe CF phenotype, and people with this condition will require a wide variety of medical interventions and therapies throughout their lives to address the symptoms of their condition. CF affects many different organ systems, but the most serious consequence of the disease is degeneration of lung function due to chronic respiratory infection and colonization of the airways with opportunistic microbial pathogens. Improvements in therapeutics, particularly the effective use of antibiotics, have led to significant gradual increases in life expectancy. There remains, however, a continuing need for newer, safer and more effective antimicrobials and mucolytic agents to maintain and improve our ability to combat CF lung infections before other curative approaches which target the root cause of the disease become available.

**Figure 1.**
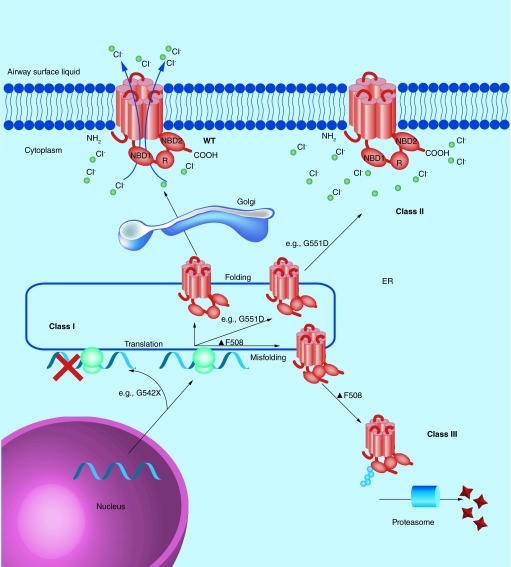
**Class I mutations (such as G542X) lead to the premature termination of CFTR protein translation.** Class II mutations include the common F508del (or ΔF508) which lead to the misfolding of CFTR protein and subsequent polyubiquitination and destruction by the cell proteasome. Class III mutations (such as G551D) are also misfolded, but may be transported to the plasma membrane. They are either poorly regulated or nonfunctional and are subsequently degraded by the cell. Class IV mutations lead to a receptor with reduced chloride conductance, whilst class V mutations lead to reduced expression levels of CFTR. Class VI mutations lead to a higher turnover of CFTR at the plasma membrane. Class IV, V and VI mutations lead to a nonclassical or atypical CF phenotype and are not shown.

## Cystic fibrosis

Cystic fibrosis (CF) is an inherited, multiorgan, multifactorial protein misfolding disease with its major pathologic impact being on respiratory function. Digestive, reproductive and other co-morbidities are also common in CF patients; a life-shortening disease that affects around 1 in 2500 babies of Caucasian ethnicity. Symptoms of the condition vary between individuals, but historically this condition was associated with mortality in infancy. Today, with improvements in the management of the condition, and application of the range of treatments and protocols required for management of the CF patient, many of those affected live into their 40s or beyond. Other factors in improved life expectancies are the recent development of novel, personalized medicinal therapies and advances in transplant surgery such that a child born with CF in the UK today can expect to live into the 5th or 6th decade of their life [1]. Here we discuss the genetic and protein-level basis for cystic fibrosis, current treatments and latest developments in the treatment of this condition.

The vast majority of affected individuals will have a mutation in the *CFTR* gene, encoded on human chromosome 7. Cystic fibrosis is an autosomal recessive disease but the spectrum and nature of CF symptoms are largely dependent upon the type of mutation(s) and their interactions. It is often described as a heterogeneous genetic disorder and genotyping of CF patients is the basis of tailored genetic counseling, epidemiological studies and the development of personalized medicine (i.e., mutation-specific therapies combined with symptom specific supportive treatments/care). The ‘sweat test’ which determines secreted chloride levels in sweat (abnormal levels being a hallmark of the condition) and antenatal ultrasound are the nonspecific diagnostic approaches currently employed for CF [2].

Over 1000 different mutations in the *CFTR* gene have been associated with cystic fibrosis, but some mutations are more common than others and there are ethnic and regional preponderances. Mutations can be classified by the way they alter the fate of the CFTR protein. There are currently six recognized classes of mutation, and three of the most common are illustrated in Figure 1. The fully functioning wild-type protein is a transmembrane ion channel with two membrane-spanning domains (MSDs) which span the membrane six times. It has two nucleotide binding domains (NBDs) and a regulatory domain (R) and transports chloride (and other anions) along the electrochemical gradient. Mutations of the *CFTR* gene lead to either misfolding and consequent degradation or dysfunction/altered expression of the *CFTR* protein or can also prevent translation of the CFTR protein.

Protein misfolding of the CFTR results in to a buildup of intracellular chloride ions which is thought to draw in sodium ions and water down electrochemical and osmotic gradients. One theory is that this leads to dehydration of the surface airway fluid and creates the thick, sticky mucous seen in individuals with CF, and that this creates an environment susceptible to colonization with opportunistic pathogens. Although this was the prevailing theory for many years it is likely that defects in CFTR function also have effects on innate immunity at the airway epithelial cell surface. CFTR also transports thiocyanate ions (SCN^-^), which are a key component of the innate immune defense. Thiocyanate and hypothiocyanite (OSCN^-^) are able to react with potentially damaging innate immune mediators and are thereby thought to have a protective role in protecting the lung during inflammation which may be absent in people with CF [3].

CFTR misfolding also impact the digestive and reproductive systems. People with CF can have gastrointestinal symptoms associated with malabsorption of dietary nutrients, particularly fats, due to reduced enzyme secretion in the pancreas. Some of these symptoms can be addressed through diet management and the use of enzyme replacement therapies. In the reproductive system, some men with mutations in *CFTR* can have bilateral absence of the vas deferens. Although these affected individuals are usually fertile (i.e., they do produce viable sperm) they would require assisted reproductive technologies to reproduce as the vas deferens have usually degraded prior to birth due to clogging with thick mucus. Although these symptoms will have significant impacts on the quality of life of CF patients, it is the lung infections with opportunistic microbial pathogens such as the bacteria *Pseudomonas aeruginosa* and *Burkholderia cepacia* and the fungi *Aspergillus fumigatus* and consequent respiratory degeneration and failure which are the ultimate cause of death in most individuals with CF. Repeated infections over the duration of their lifetime has an impact upon lung capacity and architecture. Improved physiotherapy regimes, infection prevention strategies and antibiotic therapy have had some of the biggest impacts on improved life expectancy, but the ultimate aim as a means to a cure versus treatment would be to restore the functionality of the misfolded CFTR in CF patients.

## Class I, II & III mutations & protein folding & function

A wide variety of mutations in *CFTR* have been characterized which have been linked with the cystic fibrosis phenotype. As described above (Figure 1), these can be categorized based upon the impact of the mutation on the fate of the CFTR protein. People who are homozygous for class I, II and III mutations most often have a severe cystic fibrosis phenotype and some examples of these classes of *CFTR* mutation are explored here in more detail. Class IV, V and VI mutations often leave some residual CFTR function and therefore have a less severe phenotype.

F508del-CFTR, or delta-F508 (ΔF508), is the most common *CFTR* mutation leading to cystic fibrosis [4] F508del-CFTR is a class II mutation. A deletion of three nucleotides in the gene leads to the deletion of the phenylalanine residue at position 508 of the polypeptide chain. Although the protein is fully translated, the absence of this phenylalanine residue leads to misfolding of the CFTR protein in the endoplasmic reticulum and the protein is subsequently degraded. Perhaps surprisingly, wild-type CFTR is also subject to some degree of proteasomal degradation at this stage, but a proportion of functioning wild-type CFTR protein makes it to the plasma membrane allowing for normal chloride ion transport, and this is not the case in people homozygous for F508del-CFTR. The cell proteasomal machinery and the action of ubiquitinases were found to be essential for the recognition and rapid degradation of the misfolded F508del-CFTR protein [5]. A chain of ubiquitin molecules, covalently linked together at Lys48, are needed for the recognition of a labeled protein by the cell proteasome. This phenomenon is known as polyubiquitination [6]. One of the therapeutic goals in cystic fibrosis has been to develop drugs which can assist the correct folding of F508del-CFTR to bypass this cellular quality control step and to restore some function.

Class I mutations (such as G542X) are thought to be the genetic basis for as many as 10% of CF cases [7]. This class of mutation is defined as impairing the translation of the protein and is caused by the creation of premature stop codons leading to the production of truncated proteins and consequently no functioning CFTR at the plasma membrane. In this specific case the codon for glycine residue (G) at position 542 on the polypeptide chain has been mutated from GGA to the stop codon TGA [8].

Class III mutations, such as G551D, also results in some changes to the structure and function of the CFTR protein. This specific mutation is a missense mutation which leads to an aspartate (D) reside at position 551 instead of the amino acid glycine (G). This effectively swaps a small nonpolar amino acid for a negatively charged amino acid. It is the most common specific mutation in this rare class, and results in a severe CF phenotype. Although the CFTR protein is not removed by cellular quality control systems and is trafficked to the plasma membrane, the mutation leads to the abolition of ATP-dependent gating of the ion channel. This means it is much more likely that channel will be in a ‘closed’ conformation when compared with wild-type channels and it leads to impairment in anion conductance [9].

## Unmet medical need – an orphan disease

Cystic fibrosis is defined as an orphan disease in that it affects fewer than 200,000 individuals in the USA and has a similar incidence elsewhere in the developed world. The US Orphan Drug Act of 1983 and equivalent legislation worldwide (Australia, Japan and Europe predominantly) provides regulatory and commercial incentives to develop treatments for rare/ultrarare diseases to counterbalance the low numbers of patients requiring medicinal treatments. Orphan drug schemes were introduced to address the very low levels of R&D activity in the rare disease space and allowed for the creation and success of a number of highly innovative biotechnology companies who have come to dominate the orphan drug discover space which ‘big pharma’ shunned until recently in favor of mass-market ‘blockbuster’ drug discovery and development strategies.

Despite the range of medical interventions CF patients depend on to maintain any quality of life, there are a very limited number of targeted, specific CF therapies and there had been very little progress as regards the discovery of new treatments until very recent times. Despite the market incentives orphan schemes provide and a recent resurgence of R&D efforts in this space, CF remains a highly complex, challenging clinical problem to solve and in the absence of a cure, there remains a need to develop more effective, long-term therapeutics to tackle various aspects of the condition; a priority being well-tolerated, resistance-free medicines for treating and preventing infection in the airways of CF patients and as a consequence improving lung function.

In addition to commercial efforts to develop new CF treatments, research into rare diseases (as well as patient support and disease awareness) in the UK is also supported through research councils UK (RCUK) and charitable trusts like the Cystic Fibrosis Trust, and Rare Disease UK (RDUK). Funding supports projects seeking to understand the fundamental mechanisms of CF and molecular epidemiology, from blue-skies research to applied science which target the development of new therapies. Seed funding, such as that provided by the National Organization for Rare Disorders (NORD) in the USA has been used to develop new research ideas into commercial possibilities, which have also been supported by large US charities such as the Cystic Fibrosis Foundation. CF research will continue in both the academic and commercial sectors, but in the commercial sectors many of the recent developments in CF therapeutics have come from smaller biotechnology firms, in line with trends in orphan drug discovery activities overall.

## Current treatment options

There is no cure for CF, and treatments largely address multiple symptoms and not the underlying cause of the disease (i.e., protein misfolding). There are a wide range of medical interventions required for CF patients (summarized in Table 1) which have to be tailored to the individual patient and which depend on their specific symptoms, disease etiology, age and general health.

The most successful therapeutic intervention in terms of reducing the morbidity and mortality in CF has been the use of antibiotic therapy for the treatment of CF associated lung disease. Inhaled antibiotics such as tobramycin [32] and colistin [33] have had a significant impact on mortality [34]. Inhalation minimises the systemic side effects of long term antibiotic use.

Many of the treatments described above target respiratory symptoms as infection leads to a gradual deterioration in lung function in CF patients. Bilateral lung transplant surgery is only suitable for a limited number CF patients when their lung function has been badly affected. There is a shortage of donor organs and there are inherent risks with major surgery. CF patients who are evaluated for this procedure would also need to be on anti-rejection medication for life. Antibiotic therapy is often necessary to optimize the surgical outcome, and colonization with certain microbes would preclude surgery.

## Recent developments in treatments which target the underlying causes of CF

A few recent advances in CF therapies address the underlying causes of CF. Kalydeco™ (Ivacaftor/VX-770; Vertex, MA, USA) was approved by the FDA in 2012 as a treatment for people with the CF class III mutation G551D. This rare gating mutation, discussed above, has also been nicknamed the ‘Celtic’ mutation because of its prevalence in NW Europe, with Ireland having the highest prevalence of G511D CF at 5.7% [11]. Ivacaftor (VX-770), has been demonstrated to improve lung function in people with at least one G551D allele [12]. It is thought that VX-770 directly binds the CFTR protein and alters the regulation of the protein in a phosphorylation-dependent manner, which increases the likelihood that the channel will be in the ‘open’ conformation, therefore restoring some function [13]. Phase III clinical trials have demonstrated a 10.7% increase in forced expiratory volume (FEV_1_) measurements of lung function in comparison to controls, and an improvement of 14.2% in FEV_1_ measurements over the course of the trial.

Another Vertex (Boston, USA) compound, lumacaftor (also known as VX-809), is in development as a treatment for the much more abundant F508del-CFTR class II mutation. This compound partially corrects some of the protein misfolding so that some of the protein escapes endoplasmic reticulum-associated degradation and is translocated to the cell surface. *In vitro* experiments have demonstrated some restoration of chloride transport across the membrane is restored in cells treated with VX-809, and it is thought that this compound works by stabilizing the conformation of membrane-spanning domain 1 (MSD1) [14]. Further *in vitro* experimental data on lung cells showed that combination of VX-809 treatment with the potentiator VX-770 (Ivacaftor) enhanced chloride transport [15]. Phase IIa clinical trials using lumacaftor alone demonstrated safety and tolerability of the compound, but failed to improve clinical outcomes measured by FEV_1_ [16], therefore, Phase III clinical trials in 2014 examined treatments with lumacaftor alone and in combination with ivacaftor. Results from this trial were published very recently and demonstrate improvements in treated versus predicted FEV_1_ of 2.6–4.0% and reduced exacerbation rates [31]. The impact of the drugs in the target patients population seems to be greater on minimizing exacerbations than FEV1 so distinct from the monotherapy approach with ivacaftor alone. A US FDA approval decision for the combination therapy is expected in July 2015. Although it remains to be seen if early intervention with any of the potentiator/corrector therapies will rescue lung function and reduce morbidity in the long term, these treatments do provide hope along with other supportive therapies, treatments that tackle the folding and function of the CFTR protein might extend the lives and reduce morbidity for people living with CF.

Ataluren is another small molecule in development as a potential therapeutic for certain CF genotypes that targets the CFTR. It is being developed by PTC therapeutics and may also be a potential treatment for some people with Duchenne muscular dystrophy and some other rare conditions as it may overcome problems caused by mutations which lead to premature stop codons, (or nonsense mutations) [17]. It is thought to work by forcing the read-through of transcripts of genes containing a nonsense mutation and has been the subject of Phase II clinical trials in adults and children with rare class I mutations of CF such as G542X (described above) which have demonstrated some improvements in chloride transport in sinus membranes and improved some clinical parameters. It is orally bioavailable and has potential to treat a number of genetic conditions caused by nonsense mutations [18].

Other pharmacological therapies are being developed to address the symptoms and altered physiology in CF that results from CFTR misfolding. These include enhancing thiocyanate levels [19] and direct [20,21] or indirect [22,23] methods for supplementing nitric oxide in the lungs (which is reduced in CF patients [24,25]). Some groups are also investigating the possibility of promoting autophagy via pharmacological means, as this is also known to be dysregulated in people with CF [26].

Novel antimicrobials are badly needed in CF, as well as more widely in treating infectious disease, because of the well known problem of microbial resistance to current antibiotics recently addressed in a UK HM Government report chaired by Jim O'Neill [35]. The development of new antibiotics will hopefully increase the range of therapeutic options for CF patients, and those which can be used in combination with other drugs to reduce the development of antibiotic resistance. Combinatorial antibiotic therapy in CF is in development, such as recent clinical trials using inhaled fosfomycin together with tobramycin [36]. Individual or combined therapies which tackle more than one of the consequences of CF, such as co-delivery of DNAseI with Ciprofloxacin [37] could also prove to be successful. Lynovex^®^, is a compound in clinical development by NovaBiotics Ltd (Aberdeen, UK) and has potential to be used as an adjunct therapy in CF as it has been demonstrated to have antibacterial, antibiofilm and mucoactive activities [38]. Also existing antibiotics, such as aztreonam [39] and levofloxacin [40] have been reformulated for inhalation and have been, or are being, re-purposed for use in treating CF lung disease. Inhaled macrolides [41], such as azithromycin, are also being used primarily because of potential anti-inflammatory [42] and anti-virulence [43] properties secondary to their well-known antimicrobial activity as this is thought to be less important when many common CF pathogens such as *P. aeruginosa* are regularly found to be resistant [44].

The therapy candidates currently in development and those recently approved represent important milestones in CF management, but these pharmacological interventions have not yet been demonstrated to be wholly curative. An alternative approach which has been also been a focus of research, is gene therapy. In theory, replacing a faulty gene in all the affected cells with a stably expressed healthy gene could be curative for CF lung disease. Obstacles to gene therapy in CF include the heterogeneous genetic nature of the disease, difficulties in transfecting lung tissue, finding suitable and tolerable genetic transfer agents or vectors, and obtaining stable expression of the wt CFTR protein. Although there have been a number of preclinical and a few clinical developments with regards to gene therapy for CF, Cochrane systemic review analysis of the literature do not yet support topical gene therapy as a treatment for CF [27]. Work continues, and one possible vector for use in future topical gene therapy for CF is the lentiviral vector [28]. The potential for genome editing in stem cells used for therapy in CF is also being investigated [29]. The most recent gene therapy clinical trial data examined nebulised liposomal delivery of plasmid DNA into the lungs of CF patients [45]. The results, although described as modest, suggested the stabilisation of lung function in CF patients receiving gene therapy compared with control with a 3.7% improvement in predicted FEV_1_.

Although limited in number, the new therapeutics under investigation for CF, including those which enable the correct folding of the CFTR protein demonstrate promise for the future. Efforts are being driven largely by the biotechnology and academic sectors from where the required innovation is being derived. As discussed elsewhere, for example by Fanen *et al.*, 2014 [30], customized and combinatorial therapeutics based upon patient genotype, and clinical need will be a major focus of future research and development.

## Conclusion

Cystic fibrosis is a rare genetic, orphan disease. The many genetic causes of the condition largely converge by the impact on the expression, or function of, the CFTR protein. Mutations have been categorised into different classes on the basis of how they effect the function of CFTR and the consequences can be mild to severe disease phenotypes. There are ethnic and geographical patterns in the frequency of different mutations, but F508del-CFTR, which causes a severe disease phenotype, is the most common, and is caused by misfolding of the protein channel. CF is a multi-organ disease, but the most serious impact in people with the severe disease phenotype, is on the respiratory system and the increased susceptibility of individuals to colonisation and infection of the airways by bacteria and fungi. The most successful treatments to date have been the use of prophylactic and inhaled antibiotics, which have treated infectious exacerbations, and managed microbial colonisation of the lungs over long periods of time, and helped to delay the progressive loss of lung function over time. Other therapeutics which have been in use for a long time also tackle the symptoms of this condition, such as the use of mucolytics to break up CF sputum. Although there have been steady increases in median mortality for people with CF, over recent decades there were very few new therapies for this condition and none of the standard drug treatments tackled the underlying cause of the disease. Only surgical intervention through transplantation had the capacity to correct the cause of the disease by replacing diseased organs. In the last few years there have been a number of promising developments, including pharmacological therapies, termed correctors and potentiators, which can correct misfolding and improve the function of the CFTR and thus target the cause of the condition.

## Future perspective

The clinical consequences of the vast majority of CF cases have been well described for decades (although there are still accounts of patients with rare mutations and atypical presentations). The physiological consequences are also now beginning to be understood: Dysregulated ion transportation across epithelial cell layers at various body sites, and the subsequent impact on body secretions is the major contributor to disease phenotype. This understanding has helped us to develop therapeutics which target the underlying cause of the disease (CFTR) and those which tackle the consequences of disease, including mucolytics, antibiotics to address respiratory infections associated with CF and digestive enzyme replacements to supplement the patient’s low secretion levels in the digestive tract.

The impact on CFTR malfunction on all aspects of innate immunity in the lung is still not fully understood or quantified. As this develops over the next few years, therapeutics supporting immunity, or replacing lost components of immunity, might make an impact in the CF field.

Advances in molecular biology have meant that the genetic component of CF is now well catalogued, and genotyping of CF patients is now well established. Our understanding of the impact of protein folding of CFTR, and potentially other proteins, as well as CF cell biology is still being fully elucidated however. Recent breakthroughs in pharmacological modification of CFTR were made through screening for active compounds, rather than rational design, but further understanding CFTR protein misfolding, cell quality control systems, and ways to modify these might lead to second and third generations of correctors and potentiators of the underlying cause of CF.

Pharmacological interventions which target the cause of CF are a breakthrough but are not curative. Earlier intervention studies in children with CF have yet to be conducted. Although even with new and improved CFTR modifiers, it is highly likely that therapies which tackle the consequences of CF will still be required. Multi-active or combinatorial therapies could tackle both the causes and consequences of CF. The struggle for new and effective antibiotics in the face of antibiotic resistance is also of increasing relevance to CF, where unavoidable chronic antibiotic use encourages resistance development and patients are susceptible to new and emerging pathogens.

**Table 1.** **A list of treatments and medical interventions employed to address cystic fibrosis.**

**Treatment**	**Frequency**	**Target**	**Purpose**
Physiotherapy	Daily^†^	Respiratory system	CPT – to help patients expectorate and improve mucocilary clearance of the airways
Enzyme replacement therapy	Regularly	Gastrointestinal tract	Helps overcome some of the problems associated with pancreatic insufficiency
PPIs and antiemetics	As required	Gastrointestinal tract	Often used to help in relieving indigestion and stomach pain and also nausea which might be a side effect of other medications
Antibiotics	As required – regularly	Respiratory system	To reduce microbial infection and colonization of the lung
Osmotic therapy	Daily^†^	Respiratory system	Hypertonic saline is usually nebulized as a sterile saline mist. Mannitol, administered as a dry powder, has also been demonstrated to improve lung function
Bronchodilators	Regularly	Respiratory system	B-adrenergic receptor antagonists (both fast acting and long lasting) are commonly used to open the airways in responsive patients and prior to physiotherapy sessions to help patients expectorate. Anticholinergic drugs are less commonly used
Corticosteroids	As required	Respiratory system	Used to ameliorate inflammation in appropriate patients with acute airway inflammation
Nonsteroidal anti-inflammatory drugs	Daily^†^	Respiratory system	There is evidence that long-term use of ibuprofen can help to delay deterioration in lung function over time, and the Cystic Fibrosis Foundation recommend its use
Mucolytic agents	Daily^†^	Respiratory system	Pulmozyme^®^, or donase alfa, is a recombinant human DNAse enzyme marketed by Genentech, and is the leading mucolytic agent used by people with CF. Extracellular DNA (human and microbial) is one of the components of the thick sticky mucous found in CF airways. N-acetylcysteine has also been used as a mucolytic agent for its effects on mucin glycoproteins
Assisted Reproductive Technologies	Rarely	Reproductive system	Can assist men who have bilateral absence of the vas deferens due to mutations on *CFTR* but no, or mild CF phenotypes. Assisted reproductive technologies such as IVF have also been used in men and women with more severe phenotypes of CF
Surgery	Once in a lifetime	Respiratory system	Bilateral lung transplants are an option in some patients with severe lung disease. Transplant surgery doesn’t cure CF (as other organs will still be affected) and it does have considerable risks but it can extend the lives of those people with CF who have very poor lung function and may improve the quality of life for some

Patients with the most common severe phenotypes will have experienced many if not most of those listed above during the course of their lives. The Cystic Fibrosis Foundation and other bodies assess the evidence of treatment efficacies gathered through Cochrane reviews and other scientific literature to make informed recommendations for CF patients and their support teams [10].

^†^Daily treatments for those who require them.

CPT: Chest physiotherapy

**Executive summary**

Cystic fibrosis (CF) is a disease caused by aberrant expression, or misfolding, of CFTR.
Mutations affecting protein folding and function: Many genetic mutations have been associated with CF, but the impact of these mutations can be classified (class I-VI) by how they affect the expression, or folding of, CFTR.
The clinical consequences of low abundance of functioning CFTR protein or of a malfunctioning CFTR can mild or very serious, and they are driven by dysregulation of ion transport across epithelial surfaces.
Unmet clinical need – an orphan disease: CF is a rare genetic condition, and within this there are people with common and rare genotypes with varying clinical symptoms. Researching and developing new therapeutics to serve this complex condition which affects relatively small numbers with regards to whole populations has required different incentives and regulatory approaches.
Current treatment options: antibiotics for CF-associated lung disease have contributed to the gradual improvement in median life-span for people with CF but new developments in this area are needed. Inhaled delivery of antibiotics is in common use and can be used in long term therapy. Other therapeutic options for reducing sputum viscocity in the lungs, such as DNAse l have also made a positive impact. Replacement enzyme therapy and dietetic input can improve nutritional status and morbidity, but surgical interventions in the GI tract are sometimes necessary. Up until recently, lung transplant was also the only way of tackling the underlying cause of CF and is still required for eligible patient's whose lung function has declined significantly.
Recent developments in treatments which target the underlying causes of CF: the CF research landscape has shifted dramatically and positively of late. After decades absent of any breakthroughs, we have seen recent advances in new therapeutic interventions that target the underlying cause and also the debilitating symptoms of this life-limiting protein-misfolding disease (e.g., inhaled antibiotics, novel CFTR corrector and potentiator therapies) and an increase in late stage CF drug research (e.g. *CFTR* gene therapy/editing) and development. These advances are significant for the CF community and also the clinical and scientific field as translation of these breakthroughs to clinical candidates/products has only come about from a much better understanding of disease mechanism and pathology.
