# Erratum

**DOI:** 10.2144/fsoa.15.57e1

**Published:** 2024-05-20

Following publication of the research article by Douglas Fraser-Pitt and Deborah O’Neil titled, ‘Cystic fibrosis – a multiorgan protein misfolding disease’, which appeared in the August 2015 issue of *Future Science OA* (*Future Science OA (Lond.)* 1(2), doi: 10.4155/fso.15.57 (2015), it has been brought to our attention that the labels for class II and class III in Figure 1 should be interchanged.

The editors of *Future Science OA* would like to sincerely apologise for any confusion or inconvenience this may have caused our readers.

